# Stem cell factor and granulocyte colony-stimulating factor reduce β-amyloid deposits in the brains of APP/PS1 transgenic mice

**DOI:** 10.1186/alzrt67

**Published:** 2011-03-15

**Authors:** Bin Li, Maria E Gonzalez-Toledo, Chun-Shu Piao, Allen Gu, Roger E Kelley, Li-Ru Zhao

**Affiliations:** 1 Department of Neurology, Louisiana State University Health Sciences Center, 1501 Kings Highway, Shreveport, LA 71130, USA; 2 Department of Biochemistry, Rice University, 6100 Main Street, Houston, TX 77005, USA; 3 Department of Neurology, Tulane University School of Medicine, 131 South Robertson, New Orleans, LA 70112, USA; 4 Cellular Biology and Anatomy, Louisiana State University Health Sciences Center, 1501 Kings Highway, Shreveport, LA 71130, USA

## Abstract

**Introduction:**

Alzheimer's disease (AD) is widely recognized as a serious public health problem and heavy financial burden. Currently, there is no treatment that can delay or stop the progressive brain damage in AD. Recently, we demonstrated that stem cell factor (SCF) in combination with granulocyte colony-stimulating factor (G-CSF) (SCF+G-CSF) has therapeutic effects on chronic stroke. The purpose of the present study is to determine whether SCF+G-CSF can reduce the burden of β-amyloid deposits in a mouse model of AD.

**Methods:**

APP/PS1 transgenic mice were used as the model of AD. To track bone marrow-derived cells in the brain, the bone marrow of the APP/PS1 mice was replaced with the bone marrow from mice expressing green fluorescent protein (GFP). Six weeks after bone marrow transplantation, mice were randomly divided into a saline control group and a SCF+G-CSF-treated group. SCF in combination with G-CSF was administered subcutaneously for 12 days. Circulating bone marrow stem cells (CD117^+ ^cells) were quantified 1 day after the final injection. Nine months after treatment, at the age of 18 months, mice were sacrificed. Brain sections were processed for immunohistochemistry to identify β-amyloid deposits and GFP expressing bone marrow-derived microglia in the brain.

**Results:**

Systemic administration of SCF+G-CSF to APP/PS1 transgenic mice leads to long-term reduction of β-amyloid deposition in the brain. In addition, we have also observed that the SCF+G-CSF treatment increases circulating bone marrow stem cells and augments bone marrow-derived microglial cells in the brains of APP/PS1 mice. Moreover, SCF+G-CSF treatment results in enhancement of the co-localization of bone marrow-derived microglia and β-amyloid deposits in the brain.

**Conclusions:**

These data suggest that bone marrow-derived microglia play a role in SCF+G-CSF-induced long-term effects to reduce β-amyloid deposits. This study provides insights into the contribution of the hematopoeitic growth factors, SCF and G-CSF, to limit β-amyloid accumulation in AD and may offer a new therapeutic approach for AD.

## Introduction

Alzheimer's disease (AD) is the major cause of dementia and the sixth leading cause of death in the United States [1]. Currently, no treatment has been proven to stop AD. Although the cause of AD remains uncertain, substantial evidence shows that toxic β-amyloid peptide plays a critical role in the progress of this devastating disease [2].

The microglial cells that cluster around β-amyloid plaques had been thought to participate in the pathogenesis of AD. Microglia activated by β-amyloid may be associated with detrimental inflammation in the brain of AD [3]. Recent studies, however, have demonstrated that microglia also have beneficial effects in AD [4,5]. Interestingly, bone marrow-derived microglia appear to be efficient in clearance of β-amyloid deposits [6].

Stem cell factor (SCF) and granulocyte colony-stimulating factor (G-CSF) are the hematopoeitic growth factors that are critically involved in regulation of blood cell production and mobilization of bone marrow stem cells [7,8]. Recently, accumulating evidence has shown that SCF [9] and G-CSF [9-11] alone or in combination (SCF+G-CSF) [9] have therapeutic effects in animal models of acute [9-11] or chronic stroke [12]. Moreover, our previous study has shown that SCF+G-CSF has stable and long-term effects to improve somato-sensorimotor function in chronic stroke as compared with SCF or G-CSF alone [12]. Further, in a short-term study, G-CSF has been shown to decrease β-amyloid deposition and to increase total load of microglia in a mouse model of AD [13].

The purpose of the present study is to determine whether SCF+G-CSF has a long-term effect to reduce the burden of β-amyloid deposits in a mouse model of AD.

## Materials and methods

All procedures were approved by the Institutional Animal Care and Use Committee of Louisiana State University Health Sciences Center and are in accordance with the National Institutes of Health Guide for the Care and Use of Laboratory Animals.

### Animal model of Alzheimer's disease

The mouse model of AD used for the present study is an amyloid precursor protein/presenilin 1 (APP/PS1) transgenic strain. This transgenic strain of AD was originally developed by Jankowsky and coworkers, who inserted two human mutant genes of AD, APP and PS1 into a single locus [14]. The genetic background of APP/PS1 mice used for the present study is C57BL/6J. These transgenic mice have been shown to develop β-amyloid deposits in the cortex and hippocampus by 6 to 7 months of age [15] (Jackson Laboratory, Bar Harbor, ME, USA). In this study, male APP/PS1 mice were used.

### Experimental design

**Figure 1:**
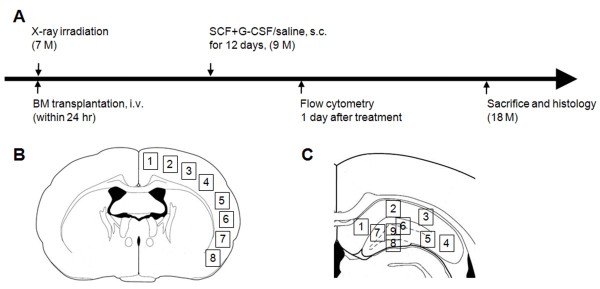
**Experimental design and image collection in the brain sections of this study**. **(a) **Schematic chart of the experimental design. Selected areas for acquiring confocal images in **(b) **the cortex and **(c) **the hippocampus. BM, bone marrow; G-CSF, granulocyte colony-stimulating factor; i.v., intravenous; s.c., subcutaneously; SCF, stem cell factor.

To track bone marrow-derived cells in the brain, 7-month-old APP/PS1 mice received X-ray irradiation to destroy their bone marrow, and the bone marrow of the mice expressing green fluorescent protein (GFP) (UBC-GFP mice with the genetic background of C57BL/6J, as with APP/PS1 mice) was transplanted to the APP/PS1 mice. Six weeks after bone marrow transplantation, mice were randomly divided into a saline control group (*n *= 5) and an SCF+G-CSF-treated group (*n *= 5). SCF+G-CSF or an equal volume of saline was injected subcutaneously for 12 days. One day after the final injection, the number of CD117-positive hematopoietic stem cells in peripheral blood was determined using flow cytometry. Nine months after treatment, at the age of 18 months, the mice were sacrificed and brain sections were processed for immunohistochemistry (Figure 1a).

### Bone marrow transplantation and hematopoietic growth factor administration

To destroy the bone marrow, APP/PS1 mice were exposed to a lethal dose of X-ray irradiation (900 rad). The ends of each femur from UBC-GFP mice (8 to 10 weeks old) (Jackson Laboratory) were clipped to expose the marrow. The bone marrow was flushed out by 10 ml ice-cold Hanks Balanced Salt Solution in a syringe with a 21-gauge needle, followed by filtration through a 70-mm nylon mesh to obtain a single cell suspension. The bone marrow cells were centrifuged, resuspended with Hanks Balanced Salt Solution, and transplanted to the APP/PS1 mice through the tail vein. Bone marrow transplantation (10^7 ^cells per mouse) was performed within 24 hours after irradiation. Mouse recombinant SCF (100 μg/kg) (PeproTech, Rocky Hill, NJ, USA) with human recombinant G-CSF (50 μg/kg) (Amgen, Thousand Oaks, CA, USA) or an equal volume of saline was administered subcutaneously for 12 days, beginning 7 weeks after bone marrow transplantation.

### Flow cytometry

Blood was collected from the tails of mice. Red blood cells were then lysed with a fluorescence-activated cell-sorting lysis buffer (BD Pharmingen, Franklin Lakes, NJ, USA) and washed with PBS containing 0.5% fetal bovine serum. The remaining cells were resuspended in PBS containing 0.5% fetal bovine serum and labeled with APC-conjugate anti-mouse CD117 antibody (anti-ckit, 1:100) or an equal amount of isotype-matched APC antibodies (eBioscience, San Diego, CA, USA) on ice for 30 minutes. The total of 5 × 10^6 ^cells was then analyzed by flow cytometry (FACSCalibur, BD, Franklin Lakes, NJ, USA).

### Immunohistochemistry

At the end of the experiment, APP/PS1 mice were transcardially perfused with PBS followed by 4% buffered formaldehyde. The brains were removed and placed in 4% buffered formaldehyde overnight at 4°C, and the brains were then cryoprotected by overnight immersion in 30% sucrose. Coronal brain sections, 30 μm thick, were cut with a microtome (Leica SM 2000R; Leica Microsystems Nussloch GmbH, Nussloch, Germany). The free-floating method was used for immunohistochemistry. Briefly, nonspecific binding was blocked by 5% normal goat serum diluted in 1% bovine serum albumin (IgG-free) (Jackson ImmunoResearch Labs, West Grove, PA, USA) with 0.25% Triton X-100. Brain sections were then incubated with primary antibodies, rabbit or mouse anti-GFP (1:200) (Santa Cruz Biotechnology, Santa Cruz, CA, USA), mouse anti-β-amyloid (1:1,000) (clone 4G8; Signet Laboratories, Dedham, MA, USA), or rabbit anti-Iba 1 (1:500) (Wako, Richmond, VA, USA) overnight at 4°C. Thereafter, the sections were incubated with Cy2-conjugated, Cy3-conjugated or Cy5-conjugated goat anti-mouse or anti-rabbit antibodies (Jackson ImmunoResearch) for 2 hours at room temperature in the dark. Sections were counterstained with 4',6-diamidino-2-phenylindole (50 ng/ml) (Sigma, St Louis, MO, USA) and mounted with ProLong Gold anti-fade reagent (Invitrogen, Grand Island, NY, USA). Brain sections without the primary antibodies served as negative controls. Four sections per each brain were processed for immunohistochemistry.

### Immunofluorescent image analysis

Images of immunofluorescent staining in brain sections were captured with a Zeiss confocal microscope (LSM510 NLO; Zeiss, Thornwood, NY, USA). Z-stacks, 16 serial optical sections with 2 μm intervals, were acquired from the cortex and hippocampus to detect the co-expression of GFP, Iba-1^+ ^cells, and nuclear dye (4',6-diamidino-2-phenylindole). Images taken from eight selected areas in the cortex and from nine selected areas in the hippocampus of each brain section (indicated in Figure 1b, c) were used for quantifying β-amyloid plaques and GFP^+^/Iba-1^+ ^cells. NIH Image J software was used for the quantitative analysis [16]. The β-amyloid load was calculated as the percentage of selected area that was occupied by the β-amyloid immunofluoresent staining. The process of cell counting was performed in a blinded manner.

### Statistical analysis

Statistical significances of changes in circulating bone marrow stem cells, bone marrow-derived cells, bone marrow-derived microglial cells and the β-amyloid deposits in the brain were assessed with Student's *t *test. The chi-square test was used for analyzing the ratio of bone marrow-derived cells appearing in β-amyloid plaques. *P *< 0.05 was considered significant, and data are expressed as the mean ± standard error.

## Results

### Stem cell factor plus granulocyte colony-stimulating factor treatment mobilized bone marrow stem cells into the blood in APP/PS1 transgenic mice

To determine whether the treatment of SCF+G-CSF mobilizes bone marrow stem cells in APP/PS1 mice, peripheral blood was collected 1 day after the final injection of SCF+G-CSF, and the levels of circulating bone marrow stem cells expressing CD117 (c-kit) were examined using flow cytometry. We found that the percentage of CD117 expressing cells was significantly increased more than seven times in SCF+G-CSF-treated mice (saline control: 1.99 ± 0.98%, SCF+G-CSF: 15.35 ± 3.0%) (*P *< 0.01), indicating that the treatment paradigm is sufficient to mobilize bone marrow stem cells into the blood in the APP/PS1 transgenic mice.

### Stem cell factor plus granulocyte colony-stimulating factor treatment decreased β-amyloid deposition in APP/PS1 transgenic mice

**Figure 2:**
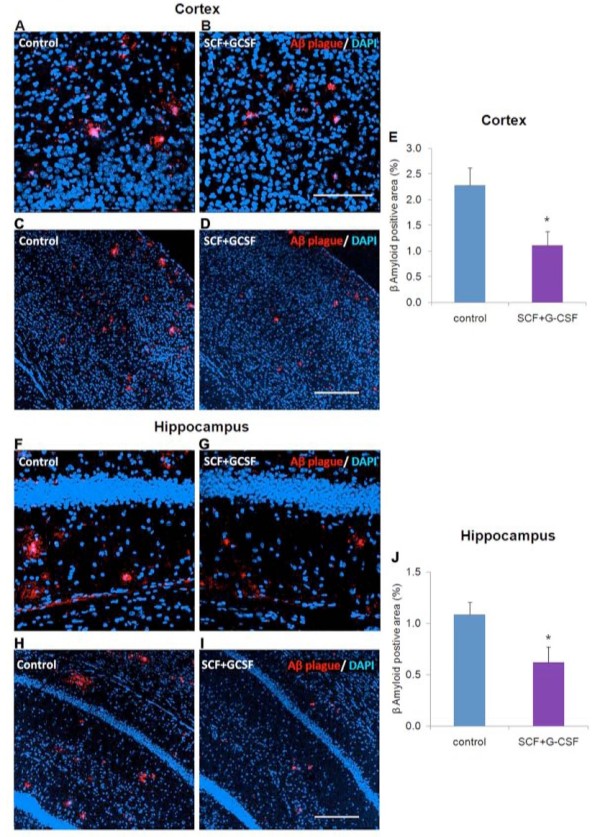
**β-amyloid deposits in the brains of APP/PS1 mice**. **(a)**, **(b) **High-power images of β-amyloid (Aβ) deposits in the cortex. β-amyloid deposits are identified with anti-β-amyloid antibody (clone 4G8) (red). **(c), (d) **Low-power images of β-amyloid deposits in the cortex. Red dots are β-amyloid deposits. **(e) **Quantification of β-amyloid deposits in the cortex. **(f), (g) **High-power images of β-amyloid deposits (red) in the hippocampus. **(h), (i) **Low-power images of β-amyloid deposits (red) in the hippocampus. **(j) **Quantification of β-amyloid deposits in the hippocampus. Note that β-amyloid burdens both in the cortex and hippocampus are significantly decreased by stem cell factor and granulocyte colony-stimulating factor (SCF+G-CSF). Cell nuclei are stained with 4',6-diamidino-2-phenylindole (DAPI) (blue). Data presented as mean ± standard error. *P < 0.05. Scale bar: (b) 100 μm (for images in (a), (b), (f) and (g)); (d), (i) 200 μm (for images in (c), (d), (h) and (i)).

Next we sought to determine whether SCF+G-CSF treatment induced a long-term reduction in the burden of β-amyloid deposits. The 12-day treatment of SCF+G-CSF started at the age of 9 months, at which the time point β-amyloid deposition in the brains of APP/PS1 mice had already occurred. The APP/PS1 mice were sacrified 9 months after the treatment, at the age of 18 months. β-amyloid deposits both in the cortex and hippocampus were examined by immunohistochemistry. We observed that β-amyloid deposits were abundant both in the cortex (2.28 ± 0.34%) and hippocampus (1.08 ± 0.12%) of control APP/PS1 mice; however, SCF+G-CSF treatment significantly reduced the load of β-amyloid deposits in these regions (cortex: 1.11 ± 0.27%; hippocampus: 0.62 ± 0.15%) (*P *< 0.05 compared with the controls) (Figure 2). These data suggest that SCF+G-CSF treatment induces a long-term reduction of β-amyloid load and that the treatment may contribute to removal of β-amyloid deposits in the brain.

### Stem cell factor plus granulocyte colony-stimulating factor treatment enhanced co-localization of bone marrow-derived microglial cells and β-amyloid plaques in APP/PS1 transgenic mice

We then asked what types of cells were involved in the clearance of β-amyloid deposits in the brains of APP/PS1 mice. The brain samples were collected 9 months after treatment. When quantifying the bone marrow-derived cells (GFP^+ ^cells) in the brain, we found there was a 1.5-fold increase in the number of GFP^+ ^cells (control vs. SCF+G-CSF: 100 ± 23.3 vs. 154.7 ± 48.9). Next we used Iba1 antibody to identify microglial cells, as Iba1 has been characterized as a specific marker for macrophage/microglia [17]. We observed that the number of GFP^+^/Iba1^+^cells in the brains of SCF+G-CSF-treated mice was twofold higher than the controls (control vs. SCF+G-CSF: 100 ± 48.6 vs. 206.4 ± 77.5). The resident Iba1^+^cells in the brain, however, did not show significant difference between these two groups (control vs. SCF+G-CSF: 100 ± 7.3 vs. 106.5 ± 8.0). These data suggest that SCF+G-CSF treatment induces a long-term effect on homing of bone marrow-derived cells into the brains of APP/PS1 mice, and that this treatment causes selective accumulation of bone marrow-derived Iba1^+ ^microglial cells in the brains of APP/PS1 mice.

**Figure 3:**
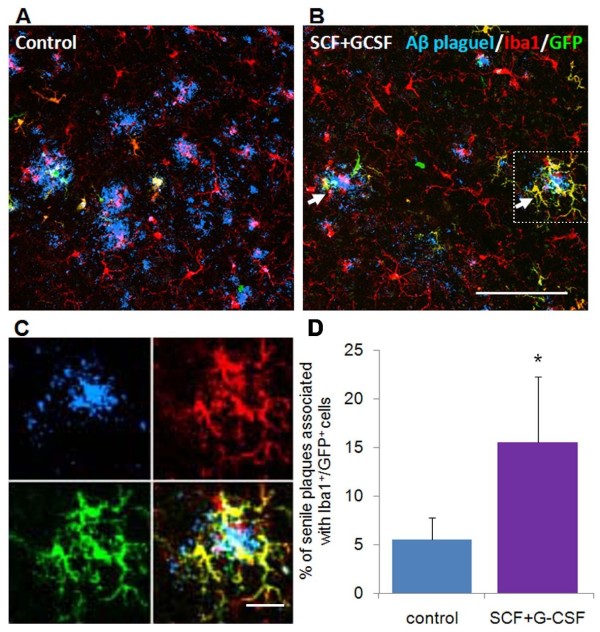
**Association between bone marrow-derived microglia and β-amyloid plaques in the brains of APP/PS1 mice**. **(a)**, **(b) **Immunohistochemical identification of bone marrow-derived microglia and β-amyloid (Aβ) plaques. Bone marrow-derived cells are the cells expressing green fluorescent protein (GFP) (green). Microglia are identified with an anti-Iba1 antibody (red). Yellow cells are the bone marrow-derived microglia that are co-labeled with GFP and Iba1. β-amyloid deposition is determined with an anti-β-amyloid antibody (clone 4G8) (blue). The white arrows indicate β-amyloid deposits surrounded by bone marrow-derived microglial cells (yellow). **(c) **High-magnification confocal images of the image indicated with dashed lines in (b). **(d) **Quantification of the bone marrow-derived microglia associated with β-amyloid plaques in the brain. Data presented as mean ± standard error. *P < 0.05. Scale bar: (b) 100 μm (for both images in (a) and (b)); (c) 25 μm. G-CSF, granulocyte colony-stimulating factor; SCF, stem cell factor.

Bone marrow-derived microglial cells have been shown to participate in removal of β-amyloid deposits in the brain [6]. We therefore sought to determine the co-localization of bone marrow-derived microglial cells and β-amyloid plaques. We performed triple immunofluorescent labeling of GFP, Iba1 and β-amyloid plauques, and found that the co-localization of β-amyloid plaques and GFP^+^/Iba1^+^cells in brains of SCF+G-CSF-treated mice (15.5 ± 6.7%) was significantly higher than in control group (5.5 ± 2.2%) (Figure 3). In other studies we have observed that the number of GFP^+ ^cells and GFP^+^/Iba1^+^cells, and the co-localization of GFP^+/^Iba1^+^cells and β-amyloid plaques in the brains of APP/PS1 mice are significantly increased by SCF+G-CSF immediately after a 12-day treatment (Li B, Liu XY, Zhao LR, unpublished obervations). These findings suggest that SCF+G-CSF treatment may enhance the effect of bone marrow-derived microglial cells on clearance of β-amyloid plaques.

## Discussion

The main findings of the present study are that systemic administration of SCF+G-CSF in APP/PS1 transgenic mice leads to long-lasting effects to reduce the β-amyloid burden. In addition, we have also revealed that the treatment enhances the co-localization of bone marrow-derived microglia and β-amyloid deposits in the brains of APP/PS1 mice.

In agreement with our present findings, G-CSF treatment in mouse models of AD has been shown to reduce the β-amyloid burden in a short-term study. Using APP/PS1 mice, Sanchez-Romos and coworkers observed that subcutaneous injection of G-CSF every other day for 3 weeks lead to acute β-amyloid reduction when examined in the third week of G-CSF injection [13]. In addition to the acute benefits, the present study shows that SCF+G-CSF treatment in APP/PS1 mice induces long-lasting effects to reduce β-amyloid load in the brain, suggesting a significant and crucial role of the combination treatment with SCF and G-CSF in AD. At present, however, how and why SCF+G-CSF treatment causes such long-term benefits in AD remains unclear. Clearly, this uncertainty will lead to many future studies to address these questions.

Mobilization of bone marrow stem cells and homing of bone marrow-derived cells in the brain are enhanced by SCF+G-CSF in AD mice. CD117, also called c-kit, is expressed on the surface of hematopoietic stem cells [18]. About 1 to 3% of peripheral blood mononuclear cells express CD117 antigen [19]. In this study, we observed that systemic administration of SCF+G-CSF in APP/PS1 mice significantly elevated CD117^+ ^cells in peripheral blood, indicating that the treatment paradigm is sufficient to mobilize bone marrow-derived stem cells. This finding is in line with other studies, which have shown that SCF in combination with G-CSF significantly increases the number of bone marrow stem cells in the peripheral blood [20-22]. It has been shown that bone marrow-derived cells can pass through the blood-brain barrier in adult brain [23]. In addition to mobilization of bone marrow-derived cells into the blood, SCF+G-CSF treatment has also been found to enhance the recruitment of bone marrow-derived cells into the adult brain [22,24]. Here we have shown that bone marrow-derived cells tended to a significant increase (1.5-fold increase) in the brains of APP/PS1 mice for a long period (9 months) after SCF+G-CSF treatment. In an acute study, however, we observed a significant increase in bone marrow-derived cells immediately after a 12-day subcutaneous injection of SCF+G-CSF in APP/PS1 mice (Li B, Liu XY, Zhao LR, unpublished observations), suggesting the ability of homing bone marrow-derived cells into the brains of APP/PS1 mice by SCF+G-CSF treatment.

The fate of bone marrow-derived cells in the brain is dependent upon the microenvironment of the brain. In our previous study, we revealed that SCF+G-CSF treatment augmented bone marrow-derived endothelial cells and neurons in the cortex of the chronic stroke brain. The number of bone marrow-derived microglia in the brain in chronic stroke, however, was not different between SCF+G-CSF-treated and mock-treated controls [22]. In the present study, we found a twofold increase in bone marrow-derived microglial cells (GFP^+^/Iba1^+^) in the brains of APP/PS1 mice 9 months after SCF+G-CSF treatment. In our acute study, a significant increase (fivefold) in the number of bone marrow-derived microglial cells in the brain was seen in SCF+G-CSF-treated APP/PS1 mice on the last day of a 12-day injection of SCF+G-CSF (Li B, Liu XY, Zhao LR, unpublished observations), suggesting that SCF+G-CSF treatment leads to an enhancement in microglial fate commitment of bone marrow-derived cells in brain with β-amyloid deposits. Other studies also provide supportive evidence that the microenvironment of the brain with β-amyloid deposits facilitates the microglial lineage determination of bone marrow-derived cells, so that injection of β-amyloid peptides into the brain is reported to cause an increase in the number of bone marrow-derived microglia [6]. Interestingly, G-CSF treatment elevated the microglial burden in APP/PS1 transgenic mice, but this treatment has no effect in wild-type mice [13].

Bone marrow-derived microglial cells participate in clearance of β-amyloid deposits. Microglia are the macrophages carrying out tissue maintenance and immune surveillance in the brain. During development, the microglial progenitors, derived from mesenchymal myeloid linage progenitor cells, migrate into the embryonic brain before maturation of the blood-brain barrier [25]. In the normal mature brain, the resident microglial cells are renewed by proliferation of endogenous microglia, whereas infiltration of peripheral monocytes into the brain is rare except in brain lesions [26]. Deposition of senile plaques is one of the pathological hallmarks of AD, and aggregated β-amyloid peptides are the major components of senile plaques. β-amyloid aggregates are clearly known to induce synaptic dysfunction and neuronal dystrophy [2].

A variety of studies have demostrated increased numbers of active microglia in the brains of AD patients and mouse models, and the microglia are associated with β-amyloid aggregates [27]. A recent study showed that, following injection of fibrillar β-amyloid into the brain cortex of aged monkeys, active migroglia mediated fibrillar β-amyloid-induced neuron loss [28]. Although it is not clear whether the neurotoxic effects are mediated by resident microglial cells or by bone marrow-derived microglia, Leung and colleagues isolated microglia from the brains of aged monkeys and revealed that fibrillar β-amyloid increased in the production of reactive oxygen species, which are toxic to neurons, in microglia [28]. In our study, we did not observe an increase in resident microglial cells in the brains of SCF+G-CSF-treated APP/PS1 mice. However, bone marrow-derived microglia in the brain were increased by the treatment. Why the treatment selectively increases the number of bone marrow-derived microglia, and whether the bone marrow-derived microglia are more beneficial than the resident microglia in the brain with β-amyloid deposits are interesting questions that should be addressed in future studies.

Accumulating evidence has recently suggested that the peripheral monocytes penetrating into the brain in AD may play a role in clearance of β-amyloid. A significantly higher number of bone marrow-derived microglia colocalizes with β-amyloid plaques, and the bone marrow-derived microglial cells effectively phagocytose and remove β-amyloid deposits from the brains of APP/PS1 mice [6]. In the present study, we found a significant reduction in β-amyloid load in the brain 9 months after SCF+G-CSF treatment. In addition, at this time point a significant increase (2.8-fold) in the co-localization of bone marrow-derived microglial cells and β-amyloid deposits was seen in the brains of SCF+G-CSF-treated APP/PS1 mice. As mentioned earlier, in our acute study, SCF+G-CSF treatment resulted in a significant increase (> 11-fold) in the co-localization of bone marrow-derived microglial cells and β-amyloid deposits (Li B, Liu XY, Zhao LR, unpublished observations). These data suggest that the SCF+G-CSF-induced long-term reduction in β-amyloid burden may be associated with a long-lasting enhancement in clearance of β-amyloid deposits by bone marrrow-derived microglial cells. How SCF+G-CSF enhances the clearance function of bone marrrow-derived microglia to remove β-amyloid, however, remains to be determined in future studies.

Both SCF and G-CSF have recently been demonstrated to be decreased in the plasma of patients with early AD [29,30]. In the present study, we have demonstrated, for the first time, a significant contribution of systemic administration of SCF and G-CSF to long-term reduction in β-amyloid burden in the brain. A combination of these two hematopoeitic growth factors, SCF and G-CSF, may thus provide a new therapeutic approach for the treatment of AD.

## Conclusions

In summary, the present study provides evidence that SCF+G-CSF treatment results in a long-term decrease in β-amyloid load in APP/PS1 transgenic mice. This therapeutic affect may be associated with SCF+G-CSF-induced increase in clearance of β-amyloid deposits through the enhancement of bone marrow-derived microglial cells. These data suggest that treatment with the hematopoeitic growth factors SCF and G-CSF may open an new avenue to develop therapeutic strategies for improving the health of individuals who suffer from AD.

## Abbreviations

AD: Alzheimer's disease; APP: amyloid precursor protein; G-CSF: granulocyte colony-stimulating factor; GFP: green fluorescent protein; PBS: phosphate-buffered saline; PS1: presenilin 1; SCF: stem cell factor.

## Competing interests

The authors declare that they have no competing interests.

## Authors' contributions

BL acquired and analyzed immunohistochemistry data and prepared the draft of the manuscript. MEG-T performed bone marrow transplantation and drug administration. MEG-T and AG contributed to maintenance and genotyping of transgenic mice. C-SP performed collection of bone marrow cells and flow cytometry. REK provided suggestions for the manuscript. L-RZ contributed to experimental design, supervision of data collection and analysis, and revision of the final version of manuscript.
